# The Tuberin and Cyclin B1 complex functions as a novel G2/M sensor of serum conditions and Akt signaling

**DOI:** 10.1371/journal.pone.0210612

**Published:** 2019-01-10

**Authors:** Elizabeth Fidalgo da Silva, Sabrina Botsford, Jessica Dare-Shih, Miranda A. Hanna, Lisa A. Porter

**Affiliations:** Department of Biological Sciences, University of Windsor, Windsor, Ontario, Canada; Univerzitet u Beogradu, SERBIA

## Abstract

A great deal of ground breaking work has determined that the Tuberin and Hamartin Complex function as a negative regulator of protein synthesis and cell cycle progression through G1/S. This is largely attributed to the GTPase activity of Tuberin that indirectly inhibits the mammalian target of rapamycin (mTOR). During times of ample nutrition Tuberin is inhibited by growth factor signaling, including direct phosphorylation by Akt/PKB, allowing for activation of mTOR and subsequent protein synthesis. It is well rationalized that maintaining homeostasis requires communication between cell growth (mTOR signaling) and cell division (cell cycle regulation), however how this occurs mechanistically has not been resolved. This work demonstrates that in the presence of high serum, and/or Akt signaling, direct binding between Tuberin and the G2/M cyclin, Cyclin B1, is stabilized and the rate of mitotic entry is decreased. Importantly, we show that this results in an increase in cell size. We propose that this represents a novel cell cycle checkpoint linking mitotic onset with the nutritional status of the cell to control cell growth.

## Introduction

Cell growth (increase in cell size and mass) and cell proliferation (more frequent cell division) are events that need to be well coordinated to maintain homeostasis and promote organismal growth and development [1,2]. Growth factor activation of the Akt/PKB pathway signals to trigger protein synthesis through the mammalian target of rapamycin (mTOR) complex 1 (mTORC1), thereby enhancing cell growth [3,4]. While this pathway is well established, specifically how the mTORC1 pathway communicates with the regulators of cell division to balance growth and proliferation remains a critical question.

Tuberin and Hamartin are highly conserved proteins encoded by the genes TSC2 and TSC1 respectively that form a heterodimeric complex known as the Tuberous Sclerosis Complex (TSC). Each of these proteins interacts with a host of important signaling pathways, however the *bona fide* functional domain of the complex is the GAP (GTPase-activating protein) domain found at the C-terminus of Tuberin [5]. In the absence of growth factors, or when nutrient conditions are poor, the TSC is activated and functions to inhibit the small G-protein Rheb (Ras homologue enriched in brain) [5]. In its inactive form, Rheb is unable to stimulate the mammalian target of rapamycin (mTOR); hence, Tuberin indirectly prevents mTOR activity under poor growth conditions. In contrast, in the presence of nutrients, Tuberin is phosphorylated by Akt [6,7,8] and ERK1/2 [9]. These modifications inhibit the GTPase activity of Tuberin, preventing inhibition of mTOR and thereby enabling protein synthesis.

Hamartin and Tuberin interact directly with proteins involved in cell cycle regulation. Hamartin interacts with Plk1, a mitotic polo-like kinase, and is phosphorylated and negatively regulated by the G_2_/M cyclin dependent kinase (CDK), CDK1 [10,11]. Tuberin negatively regulates the G1/S CDK, CDK2, by binding and altering the cytoplasmic localization of the CDK inhibitor p27 [12]. Tuberin also interacts with the G2/M cyclins, Cyclin A and Cyclin B1 (CycB1), independent of mTOR activity [13,14]. Our previous work has demonstrated that Tuberin binding to CycB1 alters the rate at which mitotic onset can occur [13]. In this current work we present data to show that Akt signaling stabilizes the Tuberin-CycB1 interaction, thereby controlling the localization of CycB1, mitotic onset, and cell growth. This represents a novel mechanism where post-translational modification of Tuberin by external signals constitutes a unique cell cycle checkpoint regulating cell growth.

## Materials and methods

### Plasmid construction

Human full-length CycB1-WT, -5xA, and -5xE in pCMX (GFP tagged) were generous gifts from J. Pines and have been previously described [15]. Full-length human TSC2-Tag2 mammalian expression vectors (flag-tagged) were generously supplied by J. DeClue. TSC2-SATA, TSC2-S939A and TSC2-T1462A were a purchased from Addgene (plasmid number 14131, 14132 and 14130 respectively deposited by Brendan Manning). TSC2-S939E was constructed using site-directed mutagenesis. TSC2ΔGAP and pCCNB1-ECFP have been previously described [13,16].

### Cell culture

Human Embryonic Kidney stock Cells (HEK293; ATCC) were maintained in Dulbecco’s Modified Eagle Medium (Sigma) supplemented with 1% penicillin-streptomycin (PS) (Sigma) and 10% fetal bovine serum (Hyclone). Cells were incubated at 37°C in 5% CO_2_ and washed with PBS and incubated with the required serum concentration (0 to 10%) for 2 hrs prior to lysis.

### Double thymidine block

Cells were transfected, incubated for 18 hr. and then medium was removed and replaced with fresh DMEM 10% FBS 1% PS. Cells were then incubated overnight in medium containing 2mM thymidine (Sigma-Aldrich). Medium was removed, cells were washed with PBS, and fresh medium added to release cells from the S-phase arrest. After 8 hrs the 2mM overnight thymidine block was repeated and followed by the PBS wash. Fresh medium with 0.5% or 10% serum was added to the cells to release from the thymidine block.

### Antibodies

Primary antibodies used were as follows: mouse α-CycB1 (GNS1; Sigma), rabbit α-Tuberin (C-20; Santa Cruz), mouse α-flag (Sigma) and rabbit α-Histone-phosphor-S10 (PH3; Abcam). Secondary antibodies used were as follows: α-Mouse Texas Red (Invitrogen), α-rabbit Alexa 488 (Invitrogen), α-mouse 568 (Invitrogen), α-Mouse IgG and α-rabbit IgG peroxidase conjugated (Sigma), α-goat IgG conjugated to agarose (Sigma).

### Immunoprecipitation and immunoblotting

Sub-confluent HEK293 cells were transfected with 5μg of DNA using Linear PEI (Polysciences, Inc.) as previously described [17]. Cells were harvested, live and dead cells counted by trypan blue exclusion, and lysed in 0.1% NP-40 lysis buffer (20mM Tris pH 8.0, 150mM NaCl, 0.1% NP-40, 1mM Na_3_VO_4_, 1mM NaF, 1mM PMSF, 1mM DTT, 10μg/ml of aprotinin, pepstatin A and leupeptin). All western blot data is reflective of representative experiments of more than 3 repeats unless otherwise noted.

For immunoprecipitation experiments, cell lysates were incubated with primary antibody (1:1000) overnight at 4°C, followed by the addition of protein G-Sepharose and incubation at 4°C for 2 hrs. These complexes were then washed with lysis buffer and resolved by 10% SDS-PAGE. The membrane was blotted with the indicated antisera (1:1000) followed by enhanced chemiluminescence (ECL) (Amersham).

Chemiluminescence was quantified on an Alpha Innotech HD2 (Fisher) and densitometry performed using AlphaEase FC software.

### Immunofluorescence microscopy

Cells were seeded onto glass coverslips and cultured as described above. Cells were transiently transfected as described above. 18 hrs following transfection, cells were fixed with 4% paraformaldehyde in phosphate-buffered saline for 2 hrs and permeabilized with 0.02% Triton X-100 for 5 min. Primary antibodies were used at 1:500 and secondary antibodies at 1:1000. Hoechst stain (Sigma) was added to the permeabilizing solution to a final concentration of 0.5μg/ml, TO-PRO-3-iodide (Invitrogen) was used as a nuclear stain at 1:1000.

### Quantification of immunofluorescence images

Cell images were obtained using a LEICA DMI6000 fluorescent microscope and data acquired using LAS AF6000 software. Cell numbers (totals and mitotic cells) were counted using Metamorph AF 1.4 software. Localization of CycB1-5xE was calculated using the number of cells staining positive for primarily nuclear or cytoplasmic GFP divided by the total number of transfected (GFP positive) cells. Cells staining both in the nucleus and cytoplasm were scored as cytoplasmic. Cells with perinuclear staining were scored as nuclear. The mitotic index (%) was calculated by scoring cells with condensed DNA or visibly undergoing mitosis and dividing by the total cells labeled with Hoechst.

Phospho-histone H3 (PH3) (%) was calculated by scoring cells positively labeled with PH3 antibody and dividing by the total number of cells labeled with Hoechst. For all applications a minimum of 10 fields of view for each slide were counted for each experiment and the mean ± SD were calculated over an average of 3 to 4 individual experiments.

### Flow cytometry analysis

Cells were transiently transfected as described above. 18 hrs following transfection cells were washed with PBS and arrested in S-phase with double thymidine block. After the blocking fresh medium with 10% FBS 1% P/S was added and cells were incubated at 37°C in 5% CO_2_ for 1–8 hrs., for starvation conditions media was changed to 0.5% serum after a 1 hr release. Cells were collected and stained with BD Transcription Factor Buffer Set for Intracellular staining (cat # 562574), using Flag primary and Alexa 488 secondary antibodies. The DNA was labelled with propidium iodide (PI). Cells were analyzed using BD Fortessa X20 cytometer. The single cell population was gated for flag expression and the flag positive (Tuberin expressing) population in G2/M phase was visualized for cell size using forward scatter (FSC-A).

### Statistical analysis

Data are presented as the mean ± standard deviation. Statistical significance was determined using Unpaired Student *t-*test http://www.physics.csbsju.edu/stats/t-test.html. *p* values < 0.05 (*), < 0.01 (**) and < 0.001 (***) were considered significant.

## Results and discussion

### Serum levels and Akt activity regulate the Tuberin-CycB1 interaction

**Fig 1:**
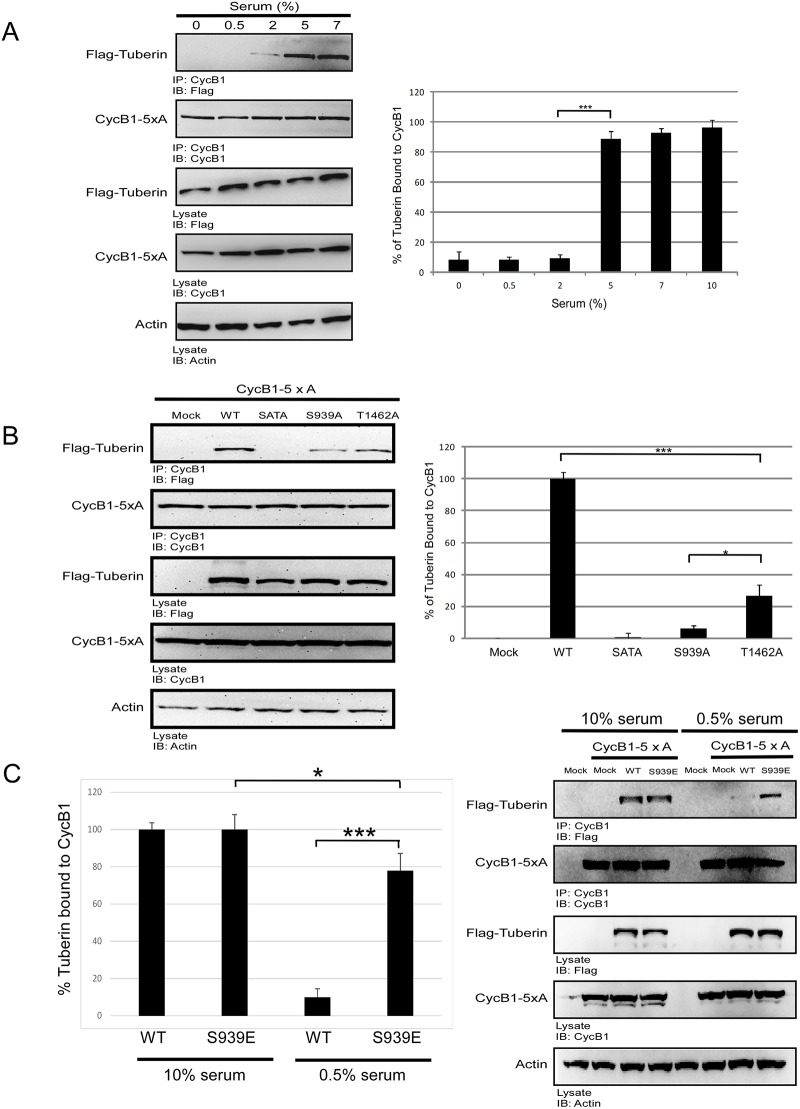
TSC2-CycB1 interaction is serum and Akt phosphorylation dependent. **(A)** HEK293 cells were transfected with Flag-TSC2 and CycB1-5xA. 18 hrs post-transfection, cells were washed with PBS and incubated for 2 hrs with different concentrations of serum (indicated as % serum). Cell lysates were subjected to IP using CycB1 antibody Representative blots; left panel. Densitometry analysis was conducted measuring the % of Tuberin bound to CycB1 (CycB1*100/Tuberin densitometry values; Y-axis) (right panel). Errors represent SD over 3 separate transfections. Unpaired t-test, *** p< 0.001. **(B)** HEK293 cells were transfected with CycB1-5xA and either pCMV-Mock control, Flag-WT-TSC2, Flag-TSC2-SATA, Flag-TSC2 S939A or Flag-TSC2 T1462. 18 hrs post-transfection cells were lysed and subjected to IP using CycB1 antibody. Representative immunoblots are shown (left panel). Densitometry analysis was conducted measuring the % of Tuberin bound to CycB1 (CycB1*100/Tuberin densitometry values; Y-axis) (right panel). Errors represent SD over 3 separate transfections. Unpaired t-test, * p< 0.05 and *** p < 0.001. (**C**) HEK293 cells were transfected with CycB1-5xA and either pCMV-Mock control, Flag-WT-TSC2 or Flag-TSC2-S939E. 18 hrs post-transfection cells the cells were incubated with 10% or 0.5% serum, 2 hrs after the cells were lysed and subjected to IP using CycB1 antibody. Representative immunoblots are shown (left panel). Densitometry analysis was conducted measuring the % of Tuberin bound to CycB1 (CycB1*100/Tuberin densitometry values; Y-axis) (right panel). Errors represent SD over 3 separate transfections. Unpaired t-test, * p< 0.05 and *** p< 0.001.

To determine whether the Tuberin-CycB1 interaction is sensitive to changes in serum levels, cells were transfected with Flag-TSC2 and a cytoplasmic form of CycB1 (CycB1-5xA) and binding studied in the presence of varying amounts of serum. The nuclear export signal in CycB1-5xA has been mutated to a non-phosphorylatable alanine to favor accumulation in the cytoplasm, this form of CycB1 has previously been shown to have a 3-fold increase in Tuberin binding over that of wild-type CycB1 [13]. The interaction between Tuberin and CycB1-5xA was significantly reduced in depleted or low serum concentrations (0–0.5%), and gradually increased as serum concentrations approached 2–10% (Fig 1A). This suggests the exciting possibility that nutrient levels can regulate the Tuberin-CycB1 interaction.

Akt is a critical regulator functioning downstream of nutrient and growth factor availability and is known to post-translationally modify the Tuberin protein on key residues S939 and T1462.. Phosphorylation of Tuberin at these sites does not disrupt the Tuberin-Hamartin interaction but are key modifications in inhibiting Tuberin GTPase function, thereby permitting mTOR activity [18]. To determine how Akt regulates the binding between Tuberin and CycB1 we mutated S939 and T1462 to a non-phosphorylatable alanine (SATA) or each site was individually mutated (S939A; T1462A) and binding assays conducted. Tuberin-SATA binding to CycB1-5xA was significantly reduced over wild-type Tuberin binding, and each of the single mutants were capable of weakly interacting with CycB1, supporting that each of these sites are important in the Tuberin-CycB1 binding interaction (Fig 1B). Mutation of T1462 retained binding to CycB1 significantly more than mutation of S939, suggesting that phosphorylation at S939 is a key site for mediating this interaction.

To test the hypothesis that phosphorylation of Tuberin by Akt can strengthen the Tuberin-CycB1 interaction, a mutant was constructed to mimic Akt phosphorylation on S939 (TSC2-S939E). Binding interactions demonstrate that this mutant has the same affinity for CycB1 as TSC2-WT in presence of serum, and rescues the binding affinity for CycB1 in low serum (Fig 1C). Notably S939E was not capable of complete rescue of binding in serum starvation conditions, indicating that additional modifications on Tuberin are important in the binding, potentially cooperation with the T1462 site. These data demonstrate that post-translational modification of Tuberin by Akt signaling enforces binding between Tuberin and Cyclin B1.

### Effects of Akt and serum levels on Tuberin-mediated retention of CycB1 in the cytoplasm

**Fig 2:**
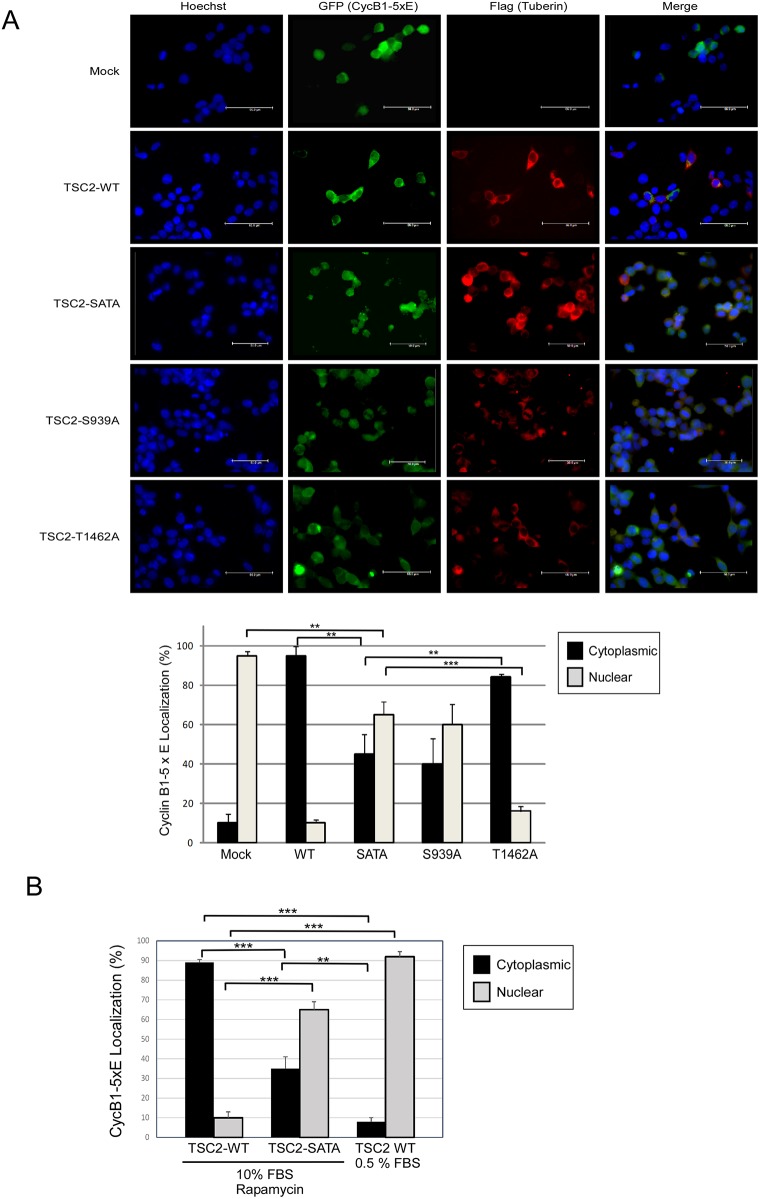
Akt phosphorylation of Tuberin regulates the localization of CycB1 and mitotic onset. HEK293 cells transfected with CycB1-5xE-GFP were co-transfected with either empty vector (Mock), TSC2-WT, TSC2-SATA, TSC2-S939A or TSC2-T1462A. **(A: top panel)** Representative immunocytochemistry showing Hoechst (Blue; first column), CycB1-GFP (Green; second column), Flag-TSC2 Texas Red (red; third column) and merge (last column). **(A: bottom panel)** Graph represents the percentage of CycB1-5xE found in the cytoplasm (solid bars) or nucleus (grey bars). Errors represent SD over 3 separate transfections. Unpaired t-test, ** p< 0.01 and *** p < 0.001. **(B)** HEK293 cells transfected with CycB1-5xE-GFP were co-transfected with TSC2-WT or TSC2-SATA. 18 hrs following transfection, cells were washed with PBS and incubated for 2 hrs with 0.5% or 10% FBS, followed by the addition of either vehicle control or Rapamycin (100nM) for 4 hrs prior to lysis. Graph shows the percentage of CycB1-5xE localized to the cytoplasm (solid bars) or nucleus (grey bars). Error bars represent SD over 3 separate transfections. Unpaired t-test, ** p< 0.01 and *** p>0.001.

The Tuberin-CycB1 interaction has been demonstrated to retain Cyclin B1 in the cytoplasm [13]. Based on our binding data, we would therefore hypothesize that elevated levels of serum, and active Akt signaling would enforce this cytoplasmic retention. To test this hypothesis we used mutants of CycB1 where the nuclear export sequence has been mutated to a glutamic acid to mimic aspects of phosphorylation (CycB1-5xE), resulting in the accumulation of CycB1 in the nucleus following import [15,19]. As we have previously demonstrated, overexpression of wild-type Tuberin retains ~90% of CycB1-5xE in the cytoplasm [13] (Fig 2A; top panel and lower left graph—mock vs. WT lanes). Interestingly, if Tuberin phosphorylation is prevented at the dual (S939-T1462; SATA) or single S939 Akt sites ~60% of CycB1-5xE moves to the nucleus (Fig 2A–lower left panel) and cells appear rounded with several demonstrating condensed chromatin and some undergoing cytokinesis. Cells were monitored by trypan blue exclusion and there is no significant difference in cell death between transfected populations (S1 Fig), supporting the potential morphological changes to the cells could be indicative of populations undergoing mitosis. Preventing the phosphorylation at T1462 had no significant difference over wild-type Tuberin. These data demonstrate that Akt phosphorylation of Tuberin on S939 is an essential modification for the functional ability of Tuberin to regulate CycB1 subcellular localization.

**Fig 3:**
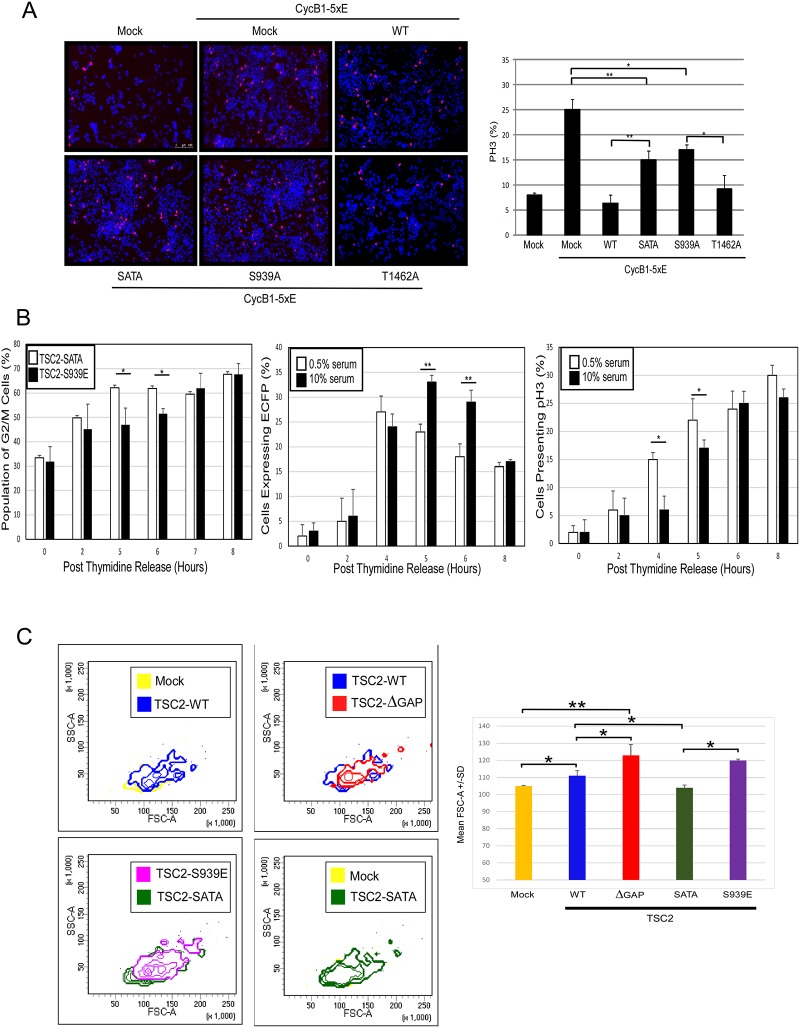
CycB1 localization and cell growth are regulated by TSC2 in mTORC1-independent fashion. **(A: left panel)** Representative images depicting Hoechst stain (Blue) and phospho-histone H3-Alexa 568 (Red) indicated as a merge. The panels are presented at 10x magnification. **(A: right panel)** Quantification of the percent of cells staining positive for phospho-H3 scored for each sample (Y-axis). Scored over 1000 cells/transfection over 3 separate transfections. Error bars represent SD. Unpaired t-test, * p< 0.05 and ** p< 0.01. **(B: left panel)** HEK293 cells were transfected with either SATA or S939E, 18 hrs after transfection the cells were arrested in S phase with double thymidine block. Cells were released from the block and collected at indicated time points for PI staining and flow cytometry analysis. (**B: middle and right panels**) 293 cells were transfected with the pCCNB1-ECFP vector, 18 hrs after transfection the cells were arrested in S phase with double thymidine block. Cells were released from the block in 10% serum and cultured in 0.5% or 10% serum and collected at the indicated time points. **(B: middle panel)** The percent of cells expressing ECFP were divided for the total number of cells staining positive for TOPRO-3 (Y-axis). Cells cultured at 10% serum (solid bars) and 0.5% serum (hollow bars) were scored over 1,000 cells/transfection. **(B: right panel)** The cells were immunoblotted with a nuclear stain (TOPRO-3), pH3 antibody. The percent of cells staining positive for pH3 are divided by total cell number staining positive for TOPRO-3 (Y-axis). Error bars represent SD over 3 separate transfections. Unpaired t-test, *p<0.05 and **p<0.01. **(C)** HEK293 cells were transfected with Mock control, TSC2-WT, TSC2-ΔGAP, TSC2-SATA and TSC2-S939E. 18 hrs after transfection the cells were arrested in S phase with double thymidine block. Cells were released from the block in 10% serum and 5 hrs after the cells were harvested and fixed in 70% cold ethanol. Transfected cells were gated using a flag immune stain and G2/M population was gated using propidium iodite DNA stain. Left panel shows Contour plots of one representative SSC-A x FSC-A spectra, forward side scatter (X-axis) used as a measure of cell size. Right panel graphic shows the mean FSC-A values obtained from 3 different transfections. Error bars represent SE over 3 separate transfections. Unpaired t-test, *p<0.05 and ** p<0.01.

We previously demonstrated that the Tuberin-CycB1-5xE interaction was mTORC1 independent [13]. Here we show that the addition of rapamycin has no effect on the ability of Tuberin WT to retain CycB1-5xE in the cytoplasm, nor did it alter the reduction in this effect seen with the TSC2-SATA mutant (Fig 3A). We further show that reduction in serum levels to 0.5% also prevents Tuberin effects on the retention of CycB1-5xE, and ~90% of cells present with CycB1-5xE in the nucleus (Fig 2B; S2 Fig). Collectively these data support that in serum starvation conditions, in the absence of Akt activation, Tuberin fails to retard CycB1 nuclear transport.

### Effect of Akt and serum levels on Tuberin-mediated delay of mitotic progression

We previously demonstrated that the Tuberin-CycB1-5xE interaction, and subsequent delay in nuclear transport of Cyc-B1, resulted in a reduction in mitotic index [13]. To determine if Akt modification of Tuberin alters mitotic onset, we measured the number of mitotic cells using PH3 (Fig 3A). All Tuberin constructs reduced PH3 staining in cells expressing CycB1-5xE, however there was a significant increase in mitotic cells when Tuberin was mutated on both (SATA) or the S939 site (S939A). Flow cytometry analysis of cells synchronized via double thymidine block and released through the cell cycle demonstrated that preventing Akt-phosphorylation of Tuberin resulted in a more rapid progression of cells into late G2/M phase of the cell cycle (Fig 3B; first panel).

To determine the effect of serum levels on the G2/M transition in the absence of manipulating the levels of Tuberin and CycB1 we transfected HEK293 cells with a G2/M reporter vector, pCCNB1-ECFP [16]. This reporter provides a fluorescent signal to time the events from late S phase to metaphase. Cells were arrested with double thymidine block (S phase arrest) and released into high serum conditions for 1 hour followed by a change of media conditions into high high serum (10%) followed by low serum (0.5%) or high serum (10%). The expression of the G2/M reporter demonstrated that low serum levels resulted in a more rapid decrease in the ECFP reporter signal, indicative of a more rapid progression into mitosis past metaphase [16] (Fig 3B–left panel). Using phosphohistone H3 (PH3) as a mitotic marker we determined that the transition between late S phase (0 hours) to mitosis is significantly delayed in the presence of high serum as compared to low serum (Fig 3B–right panel). These results support the presence of a serum sensitive G2/M checkpoint where high serum delays progression through G2 phase of the cell cycle.

### Tuberin-CycB1-mediated G2/M checkpoint regulates cell size

**Fig 4:**
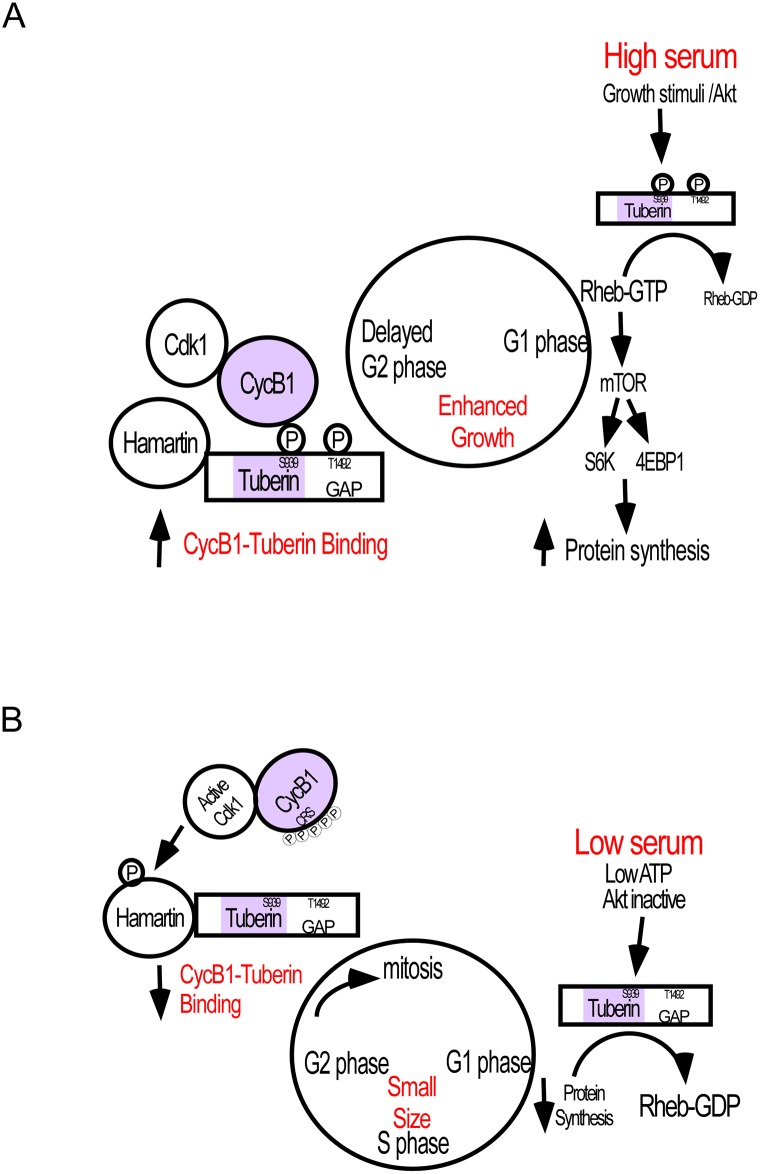
Model of Tuberin regulation of the cell cycle in response to serum status. **(Top Panel)** In the presence of serum Tuberin is phosphorylated by Akt and able to bind to CycB1 and delaying movement through mitosis, thereby enabling cell growth. (**Bottom Panel)** In the absence of serum, unphosphorylated Tuberin has reduced binding to Cyclin B1. This promotes entry of Cyclin B1 into the nucleus and mitotic progression and limits cell growth.

Petersen and Nurse have demonstrated that reduced nutrient levels in fission yeast advanced mitotic onset and cell division at the expense of cell growth [20]. We sought to determine whether the serum sensitive Tuberin-CycB1 checkpoint can control cell size in human cells (Fig 3C). Cells were transfected with flag-tagged Mock, TSC2-WT, TSC2-SATA, TSC2-S939E and a Tuberin truncation mutant lacking the functional GAP domain (TSC2-ΔGAP). The TSC2-ΔGAP is unable to inhibit mTOR and control p27 localization [21,22,23] but retains the ability to regulate CycB1 localization [13]. Approximately 18 hours after transfection cells were arrested in S-phase using double-thymidine block. 5 hours after the release cells were analyzed by flow cytometry to determine size of the G2/M population. In high serum conditions cells expressing TSC2-WT, TSC2-ΔGAP and TSC2-S939E had a significant increase in cell size, as indicated by a shift in forward scatter as compared with the Mock control and TSC2-SATA populations. Furthermore, removal of the GAP domain further increased cell size. Rosner et al. have noted that cell size regulation by Tuberin is Akt dependent, and that mutations in Tuberin that ablate the GAP domain favor an increase in cell size [24]. Our results provide a mechanistic answer to these observations. Collectively this work reveals a novel serum and Akt sensitive G2-M checkpoint that controls cell size (overview of regulation in Fig 4).

## Supporting information

S1 Fig Morphological changes to SATA cells are not due cell death. HEK293 cells were transfected with Mock control, WT-TSC2 and TSC2-SATA. 18 hrs following transfection cell were washed with PBS, fresh medium with 10% FBS 1% P/S was added and cells were incubated at 37°C in 5% CO_2_ for 24 hrs. Cells were collected and the DNA was labelled with propidium iodide (PI). Cells were analyzed in the BD Fortessa X20 cytometer. Errors represent SD over 3 separate transfections. Unpaired t-test shows no significant difference between the samples.
(TIF)

S2 Fig CycB1 is retained in the cytoplasm in an mTORC-independent manner during serum starvation. HEK293 cells transfected with CycB1-5xE-GFP were co-transfected with TSC2-WT or TSC2-SATA. 18 hrs following transfection, cells were washed with PBS and incubated for 2 hrs with 0.5% or 10% FBS, followed by the addition of either vehicle control or Rapamycin (100nM) for 4 hrs prior to lysis. Hoechst (Blue; first column), CycB1-GFP (Green; second column), Flag-TSC2-Texas Red (red; third column) and merge (fourth column).
(TIF)
