# Supplementary figures and images for: The Tuberin and Cyclin B1 complex functions as a novel G2/M sensor of serum conditions and Akt signaling
Source: PLoS One. 2019 Jan 10;14(1):e0210612. doi: 10.1371/journal.pone.0210612 (PMC6328093; doi:10.1371/journal.pone.0210612)

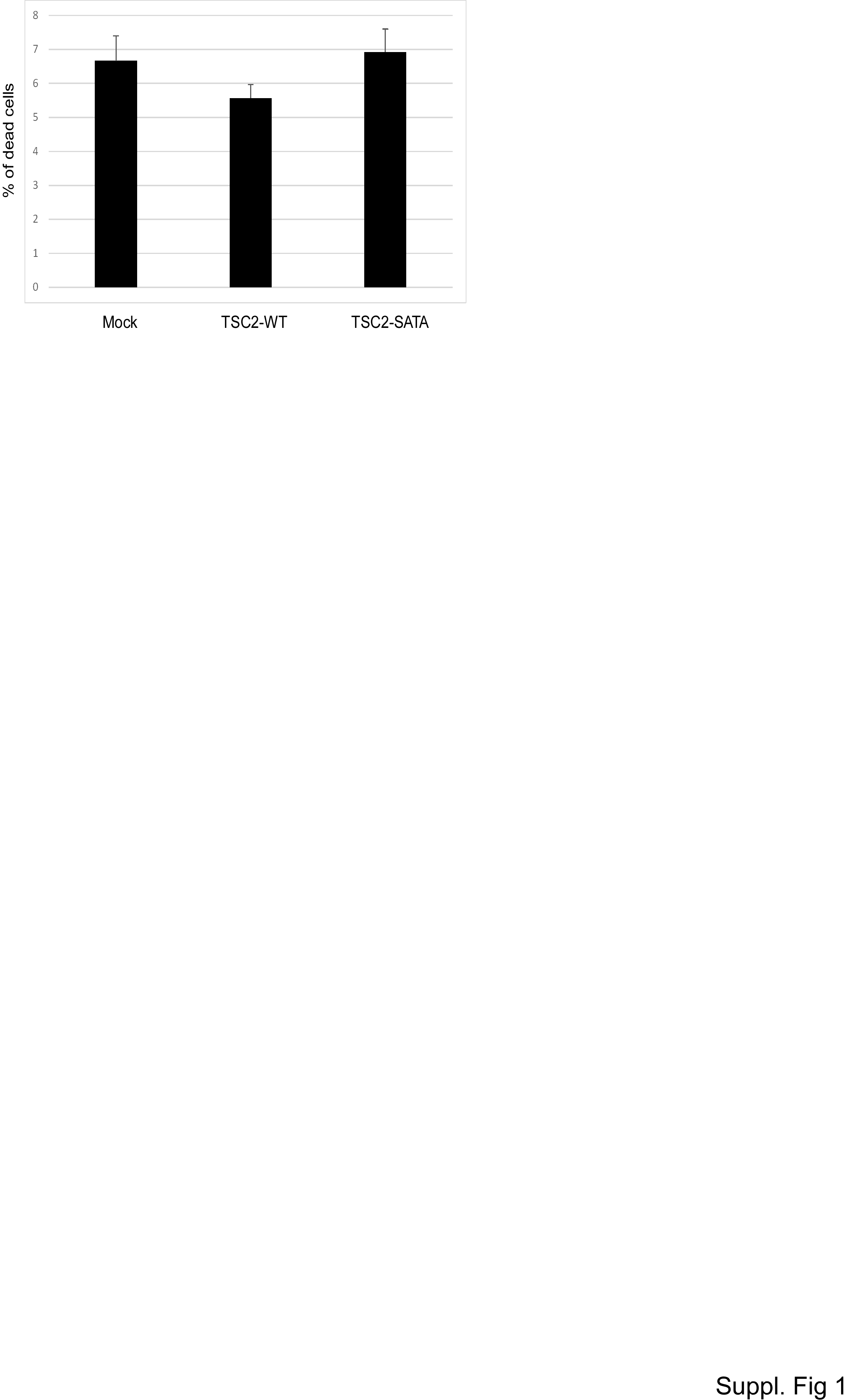

Supplement: S1 Fig — HEK293 cells were transfected with Mock control, WT-TSC2 and TSC2-SATA. 18 hrs following transfection cell were washed with PBS, fresh medium with 10% FBS 1% P/S was added and cells were incubated at 37°C in 5% CO2 for 24 hrs. Cells were collected and the DNA was labelled with propidium iodide (PI). Cells were analyzed in the BD Fortessa X20 cytometer. Errors represent SD over 3 separate transfections. Unpaired t-test shows no significant difference between the samples. (TIF) [file pone.0210612.s001.tif]

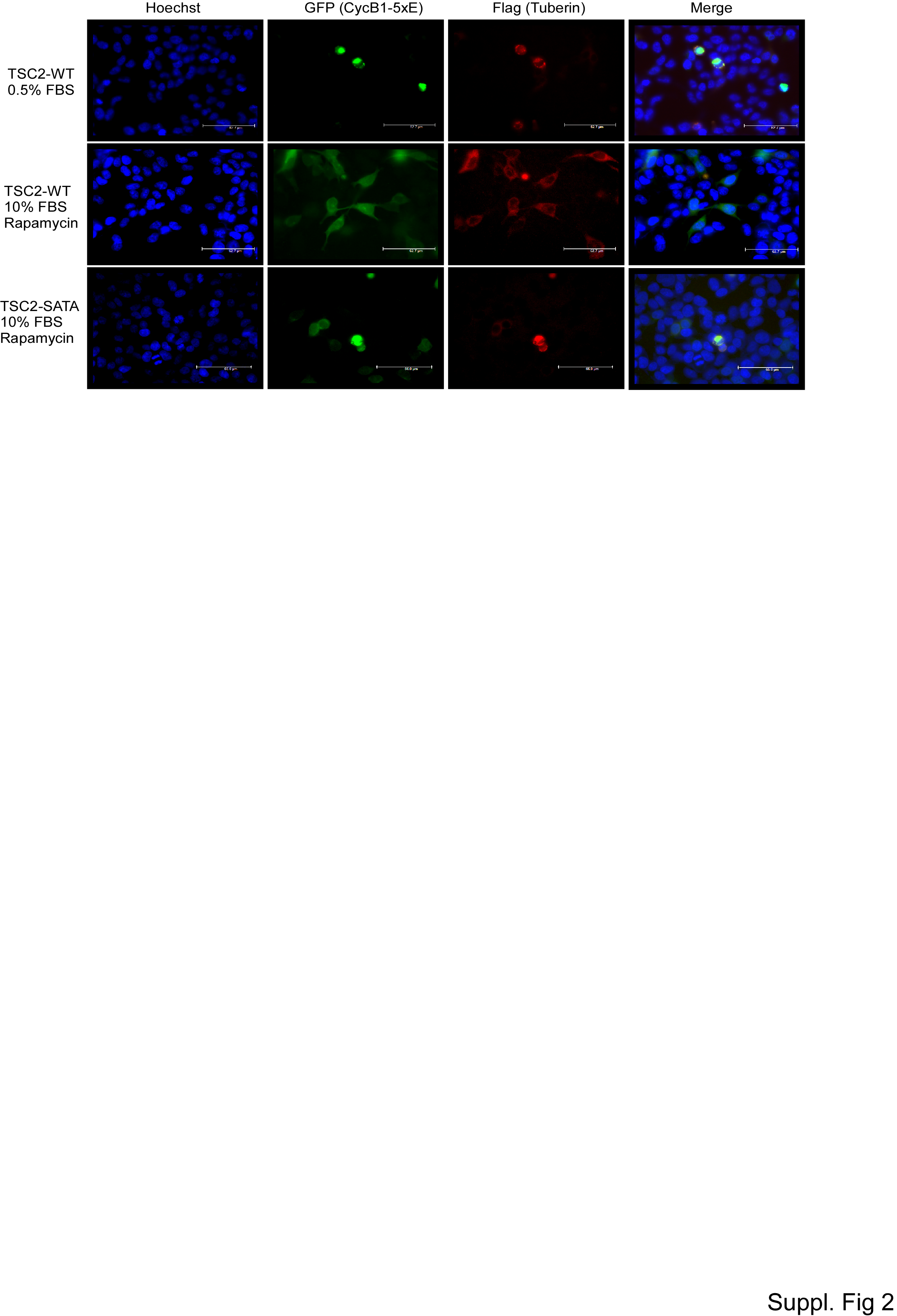

Supplement: S2 Fig — HEK293 cells transfected with CycB1-5xE-GFP were co-transfected with TSC2-WT or TSC2-SATA. 18 hrs following transfection, cells were washed with PBS and incubated for 2 hrs with 0.5% or 10% FBS, followed by the addition of either vehicle control or Rapamycin (100nM) for 4 hrs prior to lysis. Hoechst (Blue; first column), CycB1-GFP (Green; second column), Flag-TSC2-Texas Red (red; third column) and merge (fourth column). (TIF) [file pone.0210612.s002.tif]
